# Crystallization of a human galectin-3 variant with two ordered segments in the shortened N-terminal tail

**DOI:** 10.1038/s41598-018-28235-x

**Published:** 2018-06-29

**Authors:** Andrea Flores-Ibarra, Sabine Vértesy, Francisco J. Medrano, Hans-Joachim Gabius, Antonio Romero

**Affiliations:** 1 Department of Structural and Chemical Biology, Centro de Investigaciones Biológicas, CSIC, Ramiro de Maeztu 9, 28040 Madrid, Spain; 2 Institute of Physiological Chemistry, Faculty of Veterinary Medicine, Ludwig-Maximilians-University Munich, Veterinärstrabe 13, 80539 Munich, Germany

## Abstract

Among members of the family of adhesion/growth-regulatory galectins, galectin-3 (Gal-3) bears a unique modular architecture. A N-terminal tail (NT) consisting of the N-terminal segment (NTS) and nine collagen-like repeats is linked to the canonical lectin domain. In contrast to bivalent proto- and tandem-repeat-type galectins, Gal-3 is monomeric in solution, capable to self-associate in the presence of bi- to multivalent ligands, and the NTS is involved in cellular compartmentalization. Since no crystallographic information on Gal-3 beyond the lectin domain is available, we used a shortened variant with NTS and repeats VII-IX. This protein crystallized as tetramers with contacts between the lectin domains. The region from Tyr101 (in repeat IX) to Leu114 (in the CRD) formed a hairpin. The NTS extends the canonical β-sheet of F1-F5 strands with two new β-strands on the F face. Together, crystallographic and SAXS data reveal a mode of intramolecular structure building involving the highly flexible Gal-3’s NT.

## Introduction

The functional pairing of cellular glycoconjugates with tissue lectins is giving the unsurpassed structural variability of lipid/protein-linked glycans a physiological meaning^1^. In fact, reading glycan-encoded messages by these endogenous effectors appears to underlie a wide array of cellular activities^2^. At the molecular level, this pairing has a remarkably low degree of promiscuity, meaning that exclusively distinct glycoconjugates become binding partners for a particular endogenous lectin. This specificity for contact formation and of the nature of the ensuing trigger mechanisms that e.g. result in growth regulation does not only depend on mutual recognition of complementary binding sites. In addition, (i) the topological mode of glycan presentation, (ii) the lectin’s architecture and (iii) the arrangement of aggregates are emerging as factors that can contribute to the precision of the final outcome. Since respective protein families such as the C-type lectins and the ga(lactose-binding) lectins are organized in groups differing in the modular display around a common carbohydrate recognition domain (CRD)^3,4^ a fundamental importance of this type of protein design for the lectins’ activity profile can be postulated. Consequently, this hypothesis has invigorated the efforts to achieve complete structural characterization of all proteins within a family.

Looking at vertebrate adhesion/growth-regulatory galectins, three types of protein design are known: (i) the non-covalently associated homodimer (proto type), (ii) the heterodimer connected by a linker (tandem-repeat type) and (iii) the trimodular chimera, uniquely represented by galectin-3 (Gal-3)^3,5,6^. In the case of human Gal-3, the 21-amino-acid-long N-terminal stretch (NTS) with its two sites for serine phosphorylation is followed by nine non-triple-helical collagen-like Pro/Gly-rich repeats (I-IX), which harbor cleavage sites for diverse proteases^7^. The NTS and the nine repeats form the N-terminal tail (NT). In full-length Gal-3, it is connected to the C-terminal CRD that harbors two sites for c-Abl kinase-dependent tyrosine phosphorylation^7^. These three sections, i.e. NTS, collagen-like repeats and CRD (for sequences, see Supplementary Fig. S1), likely cooperate in a not yet clearly defined manner to account for this protein’s special role within the galectin network.

Inside the cell, Gal-3 can shuttle between cytoplasm and nucleus, a pathway involving import and export signals at the CRD^8–10^. Transport to late endosomes critically depends on the NT^11^. Serine phosphorylation in the NTS favors departure from the nucleus^12^. Secretion to the extracellular environment proceeds via a non-classical pathway, which appears to involve the NTS and sections of the collagen-like repeat region^13–16^. In solution, Gal-3 is monomeric, unless high concentrations are reached or bi- to multivalent ligands are present that serve as core for aggregation^17–27^. An active role in this intermolecular assembly has been attributed, in varying extents, to both the NT and the CRD^28–30^. NMR Spectroscopy data “*clearly indicate that the recombinant NT… is unstructured in solution and exists as an interconverting mixture of conformations*”, with evidence for “*significantly reduced mobility values*” in the NT region proximal to the CRD^31^. Transient intramolecular interactions occur, too. Their presence was first observed by NMR spectroscopy due to a shielding of nuclei^31^ and by electrospray ionization mass spectrometry due to a bimodal charge distribution^32^. Thereafter, NMR spectroscopy based on full-assignment work^33–35^ and studies of binding of Gal-3-derived peptides to the ^15^N-labeled CRD^36^ extended the respective body of evidence. Obviously, the NT is not an inert appendix of the CRD what explains the high interest in elucidating structural aspects of this region of the protein. In the words of a recent study, the status of our understanding of Gal-3 has been summarized that “*functional multivalency therefore is somewhat of a mystery*”^35^. For this report, we have extended crystallographic analysis beyond the CRD despite the NT’s inherent dynamics by working with a variant with shortened length of this part.

Up to now, crystallographic data are available only for a truncated protein starting at Leu^114^ as monomer^37^. No insights into properties of structurally ordered segments of the NT or a mode of CRD aggregation have so far been reported, most likely due to inherent flexibility of the full-length protein. Therefore, new routes were needed to address this issue. The stepwise deletion of collagen-like repeats from the tail by genetic engineering^38^ is a means to reduce this impediment, and, indeed, crystallization of a shortened Gal-3 variant has recently been achieved^39^. However, X-ray diffraction at 3.3 Å was insufficient for structural resolution of any part of the NT or oligomers so that further work on obtaining high-quality crystals had to be performed. Hereby, the detailed crystallographic data of ordered regions within the NT of the Gal-3[NTS/VII-IX] protein (for sequence details, please see Supplementary Fig. S1) became possible. This accomplishment led to a structural model that was set in relation to the experimentally determined shape in comparative small angle X-ray scattering (SAXS) studies of this protein, together with full-length Gal-3 and the variant with repeats IV-IX in its NT (Gal-3[NTS/IV-IX]). When combined, these investigations disclosed a conformation of two regions of the highly flexible NT seen in the crystal of Gal-3[NTS/VII-IX] and the overall shape of the three proteins in solution. Furthermore, the obtained data enabled us to suggest a molecular pattern of contacts favoring the formation of aggregates of Gal-3 into a tetramer under the given conditions driven by CRD-CRD interactions.

## Results

### Overall Folding and Quaternary Association of the Gal-3 Variant

**Figure 1:**
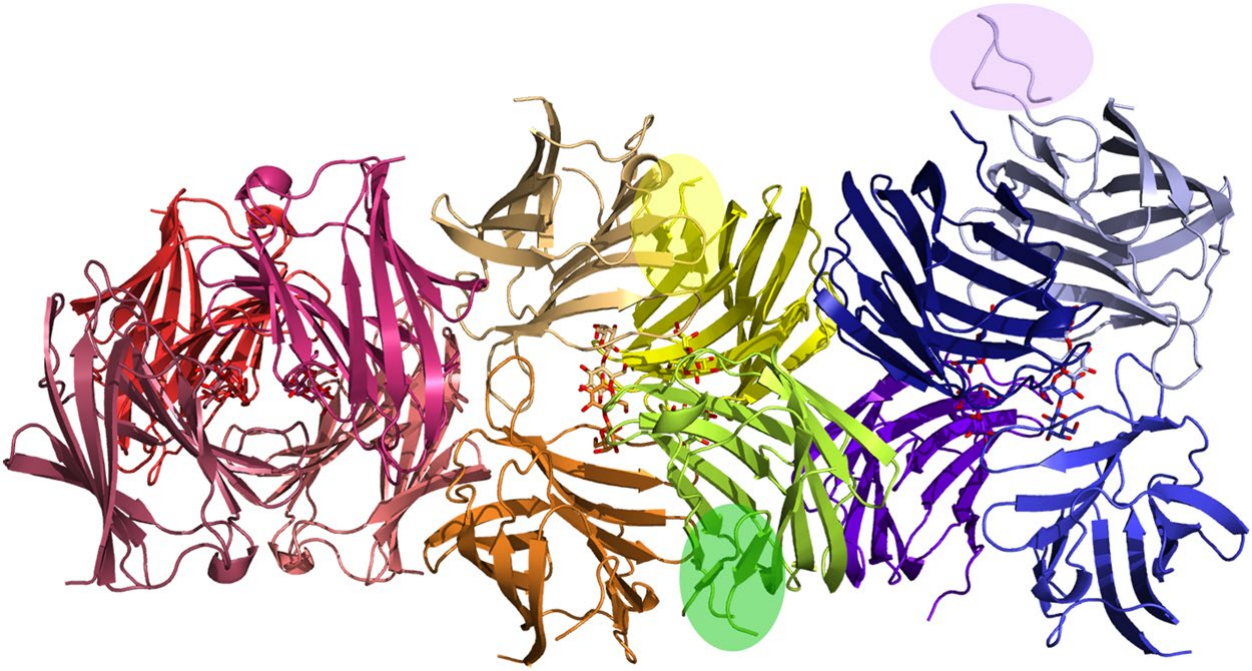
Overall folding and quaternary association. Crystal packing of Gal-3[NTS/VII-IX] in the asymmetric unit. Ribbon-diagram representation of the three tetramers with the characteristic jelly-roll topology and the CRDs facing each other. Each monomer is depicted in different colors with the bound lactose molecules in stick representation. The new segments of the NT are highlighted.

**Figure 2:**
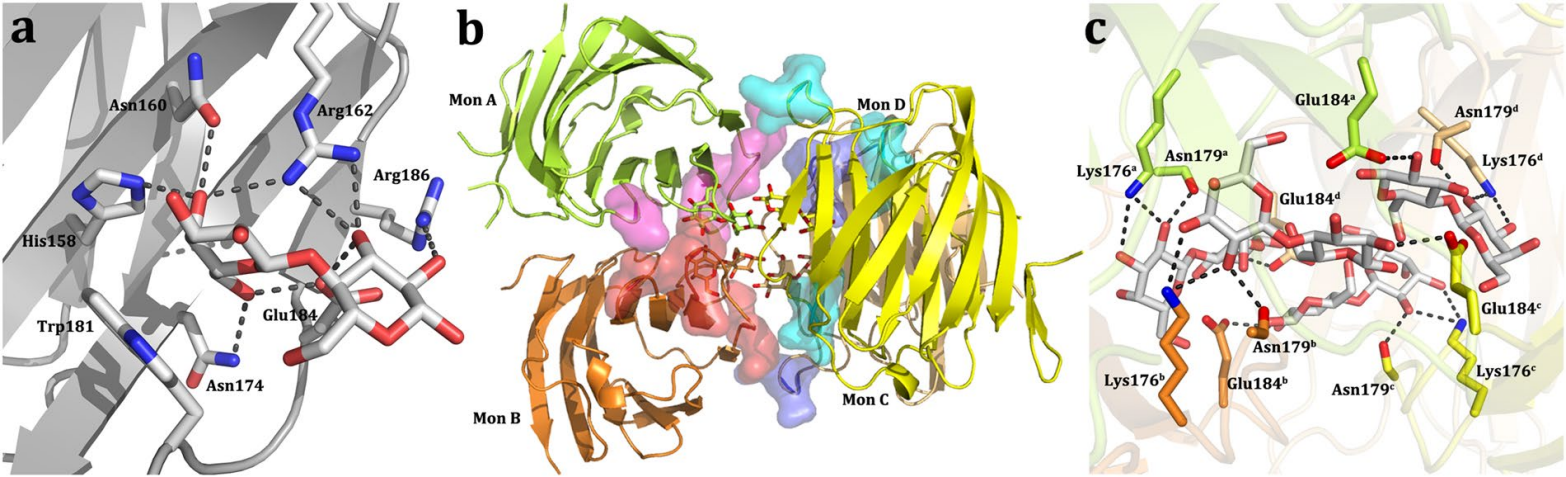
(**a**) Canonical lactose-binding site around the central Trp181. **(b)** Contacts between subunits in the tetramer. The regions involved in cross-contacts for each monomer are labeled by different colors (magenta for monomer A, red for B, cyan for C and blue for D). **(c)** Close-up view of the residues involved in the lactose-protein cross-interactions.

Crystallographic data of Gal-3[NTS/VII-IX] at 2.2 Å resolution indicated the presence of 12 monomers in the asymmetric unit (see Supplementary Table S1 for data collection and refinement statistics). These monomers are arranged as three tetramers related by two-fold non-crystallographic symmetry axes, as can be seen in the self-rotation function previously reported^39^. At the level of the monomer, the overall folding of the CRD maintains the typical β-sandwich topology of two β-sheets constituted by the antiparallel S1-S6/F1-F5 strands (Fig. 1). Lactose binds to the canonical site of each monomer (Fig. 2a).

The CRD is also the platform for homotypic aggregation. In this tetrameric arrangement, the monomers come into relative vicinity via their CRDs. Each tetramer (ABCD) is constituted by two dimers (AB and CD). The monomers of each dimer face each other by the concave surface of the CRDs, while the dimers interact by the side of the β-sandwich opposite to the NT and rotated around 90° respective to each other. The interactions at the monomer-monomer interface in each dimer (A to B and C to D) and at the dimer-dimer interface (AB and CD) are mainly hydrogen bonds. Two sets of interactions can be defined at these interfaces. One of them occurs at the respective interface (AB and CD) in a pairwise manner between Asn143 (A) and Asn153 (B) as well as between Arg162 (A) and Asn179 (B) (likewise for the CD pair). The interactions at the lateral interfaces (AC and BD) follow a similar mode of contact formation. In detail, inter-monomer contacts within the AC (and BD) interface are between Asn166 (A) and Arg183 (C) as well as between Arg168 (A) and Arg186 (C) (Fig. 2b). Other types of interactions between monomers, i.e. by protein-carbohydrate recognition, involve hydrogen bonds between amino acids of a monomer and of lactose bound to a different subunit. This type of network bridges residues Lys176 (monomer A) and Asn179 (monomer A) to the hydroxyl groups (O2 and O3) of the lactose unit in monomer B. The same type of interplay is operative in monomers C and D. Additional cross-interactions between Glu184 of monomers C and D and the O2′ from the lactose unit in monomers A and B, respectively, stabilize the tetramer (Fig. 2c). Owing to this kind of structural arrangement a channel-like cavity inside the tetramer is generated. It appears to be suited for accommodating glycans with N-acetyllactosamine (LacNAc) repeats, such epitopes are present in chains of glycoproteins such as the Gal-3 counterreceptor laminin and of glycosphingolipids such as (neo)lactotetraosyl (LNnT, LNT) ceramide.

Beyond disclosing these structural aspects of the CRD, X-ray diffraction offered the first crystallographic insights into the NT. In fact, additional electron density could be observed for regions of the NT in three of the 12 monomers. This information opened the way to describe the structure of ordered parts of this crystallographically so far uncharacterized section of Gal-3.

### Ordered Sequence Stretches in the NT

The spatial arrangement of the electron density originating from the NT indicated its assignment to two different segments of the NT rather than resulting from conformers of the same region, as graphically explained in Supplementary Fig. S2. These two sections in Gal-3’s NT were readily distinguishable. This is especially the case for the continuous electron density in one of the monomers around the start of the CRD. It could be assigned to the sequence starting at Tyr101 up to Leu114 (Supplementary Figs S1, S2a,b). In the other two instances, the electron density could unambiguously be assigned by identifying the characteristic phenyl ring of Phe5 and the imidazole ring of His8 as diagnostic indicators. Thus, the sequence from Asn4 to Pro17 in the NTS is the second source of extra electron density (Supplementary Figs S1, S2c,d). As a consequence, two parts of the NT could structurally be characterized in detail.

**Figure 3:**
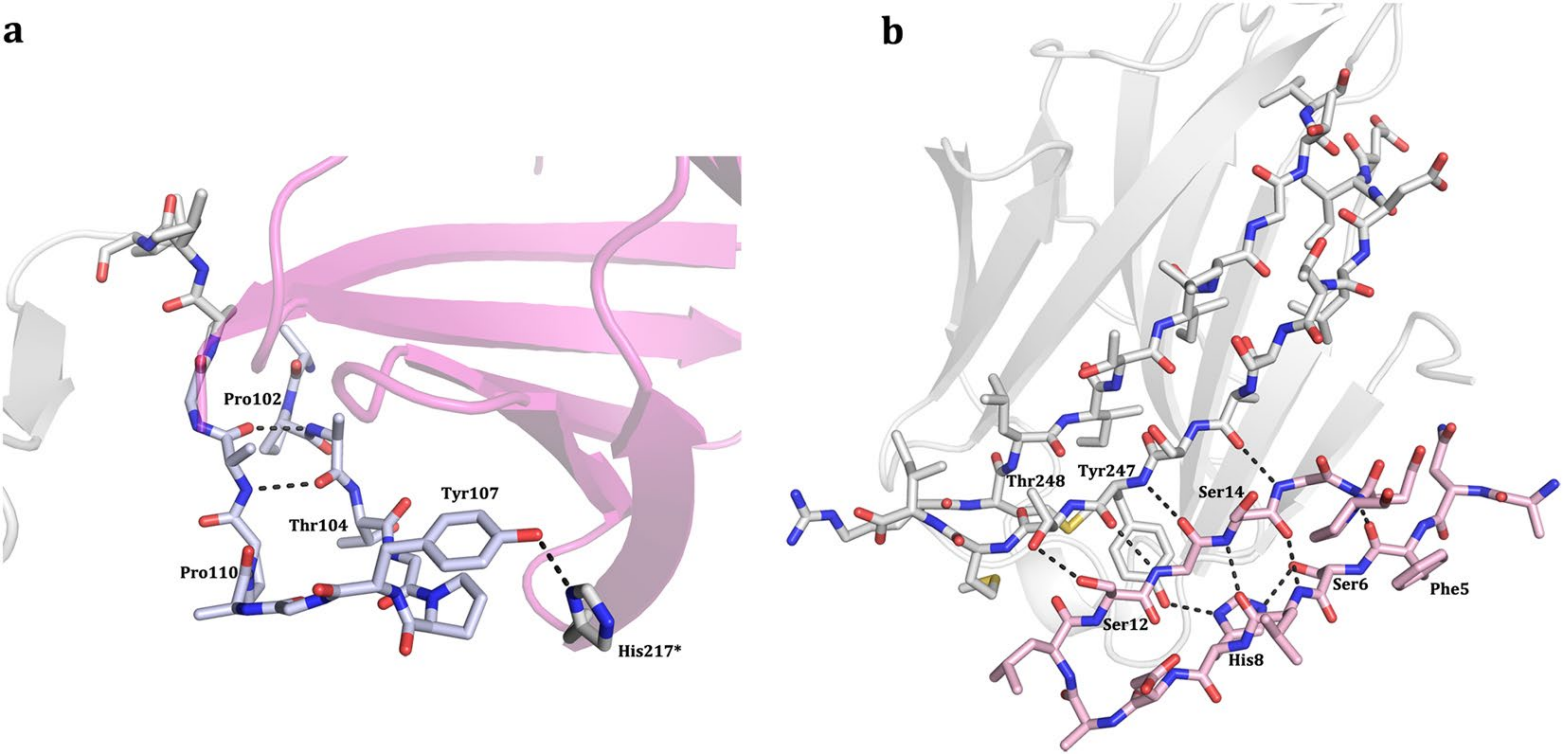
Crystallographic structure of two regions of the N-terminal tail. **(a)** Close-up view of the collagen-like repeat IX in the NT region (Tyr101-Val116). Two hydrogen bonds between Ala103 and Ala111 stabilize this section. One extra hydrogen bond with a symmetry-related molecule between Tyr107 and His217 allowed the definition of this segment. **(b)** Specific set of contacts between the C-terminal region of the CRD and NTS residues, typical of a β-sheet.

The obtained data revealed that the region in the terminal section of repeat IX and the beginning of the CRD adopted a β-hairpin structure (Fig. 3a). It is stabilized by hydrogen bonds between backbone atoms of sequential residues and also by an interaction of Tyr107 with His217 of a symmetry-related neighbor (Fig. 3a, Table 1). The proximal segment of the repeat section and the first part of the CRD thus can establish this hairpin.

**Table 1:** Hydrogen bond interactions stabilizing the N-terminal tail.

Atom 1	Atom 2	Distance (Å)
O^γ^ Ser12	O^γ^ Thr248	2.74
O^γ^ Ser12	N Asp9	2.73
N Gly13	O Tyr247	3.05
O Gly13	N Tyr247	3.04
N Ser14	O Leu7	2.71
O Ser14	N Leu7	2.76
N Gly15	O Ala245	2.76
N Asn16	O Phe5	2.53
O^γ^ Ser6	N^δ2^ His8	2.52
N Ala111	O Ala103	3.00
O Ala111	N Ala103	2.76
O Tyr107	N^δ1^ His217*	2.89

*Symmetry-related residue.

At the N-terminus, an ordered segment is detected. The NTS is arranged in a double-stranded anti-parallel β-sheet (Fig. 3b). It is stabilized by a network of hydrogen bonds (Fig. 3b, Table 1). As consequence, the residues Phe5-His8 are constituents of the first β-strand followed by the second β-strand (Gly13-Asn16), named F–1 and F0, respectively. Their presence and the observation that the F0 strand runs anti-parallel to the carboxy-terminal F1 strand combine to explain the extension of the β-sheet of the F1-F5 β-strands (Fig. 3b). The NTS appears in the crystal structure wedged between the F-faces of two separate molecules, one of them a symmetry-related molecule. The actual conformation, these two new β-strands belonging to the monomer in the asymmetric unit and not to the symmetry-related partner, was selected because the angle formed between the contacting strands is much lower (close to 0°) compared to the other conformation (approx. 45°), hereby maximizing the contact surface area and the number of contacts between the interacting strand F0 and the first strand of the F-face (F1) (Supplementary Fig. S3). In spite of our assignment and due to the inherent flexibility of the NT, this new element could belong to the symmetry-related monomer, or much less likely, to some other monomer from the asymmetric unit or a symmetry-related molecule. In any case, it is not possible to unambiguously assign the new element to any monomer, although the given assignment is the most likely scenario.

Obviously, in this spatial arrangement, flexibility of the NTS is reduced, with implications for the presentation of substrate site(s) for serine phosphorylation (please see below). Having hereby solved the structure of two segments of the NT, this information facilitated the development of a structural model of Gal-3 with an NT, albeit truncated.

### Building a Structural Model for the Gal-3 Variant

**Figure 4:**
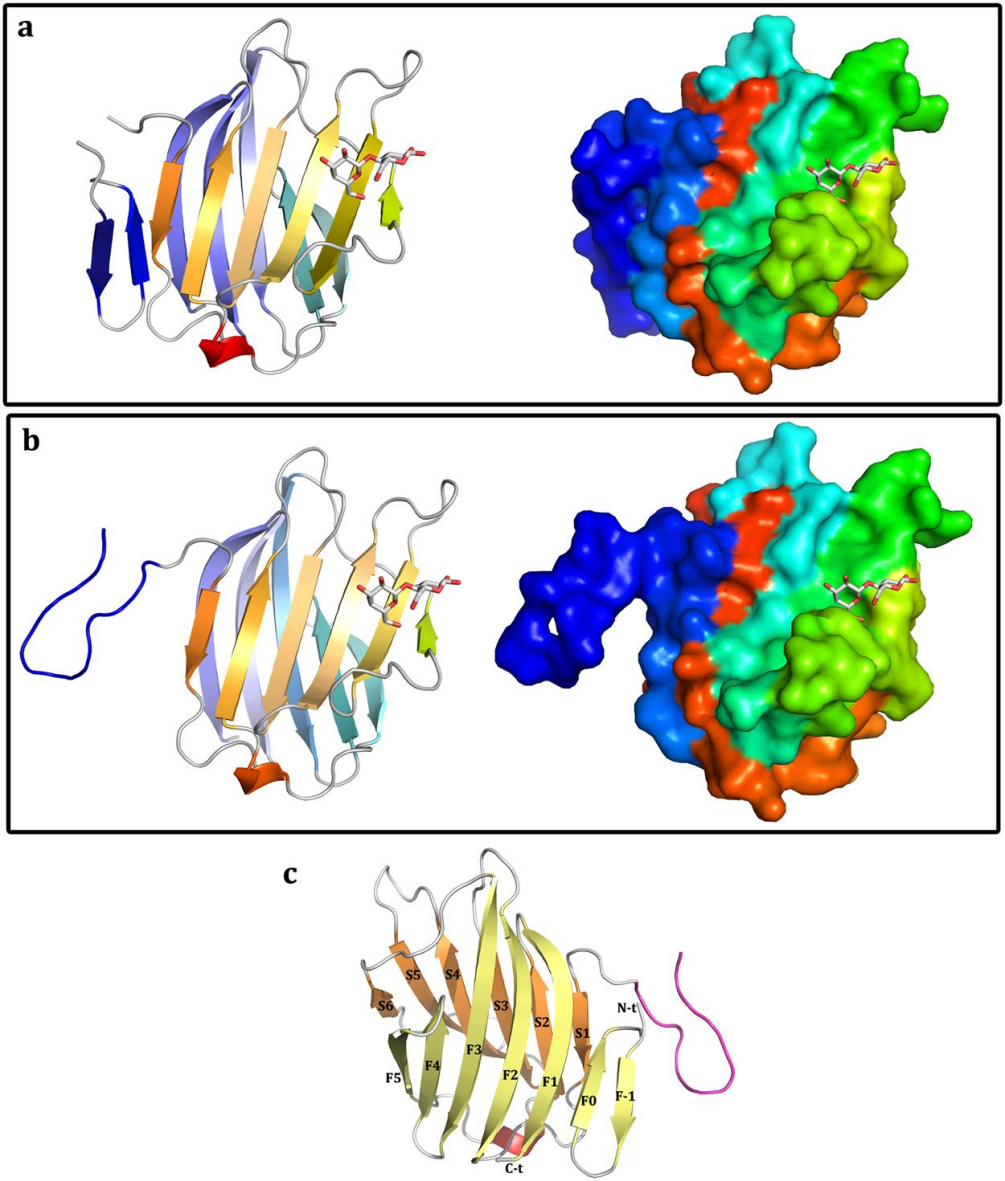
Structural model of the CRD and the two new regions described for the Gal-3[NTS/VII-IX] variant. (**a**) NTS region. Left panel: ribbon representation; right panel: surface representation; the new region shown in blue. (**b**) Repeat IX linked to the CRD. Left panel: ribbon representation; right panel: surface representation, the new region shown in blue. (**c**) Definition of the secondary structure of the CRD and the two regions defined by this study.

The two newly identified ordered structures of the NT, i.e. at its start and its terminus, provide essential information on how intramolecular recognition between NT and CRD can give shape to the full-length protein. When added to the CRD core, the NTS is able to introduce a novel double-stranded antiparallel β-sheet at the F-face (Fig. 4a). The segment with the hairpin is located at the S-face (Fig. 4b). Superposing these two separate structures a model could be generated. It served as a platform to include the remaining part of the NT as in a puzzle to build full-length Gal-3 (Fig. 4c). Overall, the core is composed of a seven-stranded β-sheet (F–1 to F5) and a six-stranded β-sheet (S1-S6) (Fig. 4c). Due to lack of electron density the repeats VII and VIII could not be modeled and likely exhibit flexibility.

**Figure 5:**
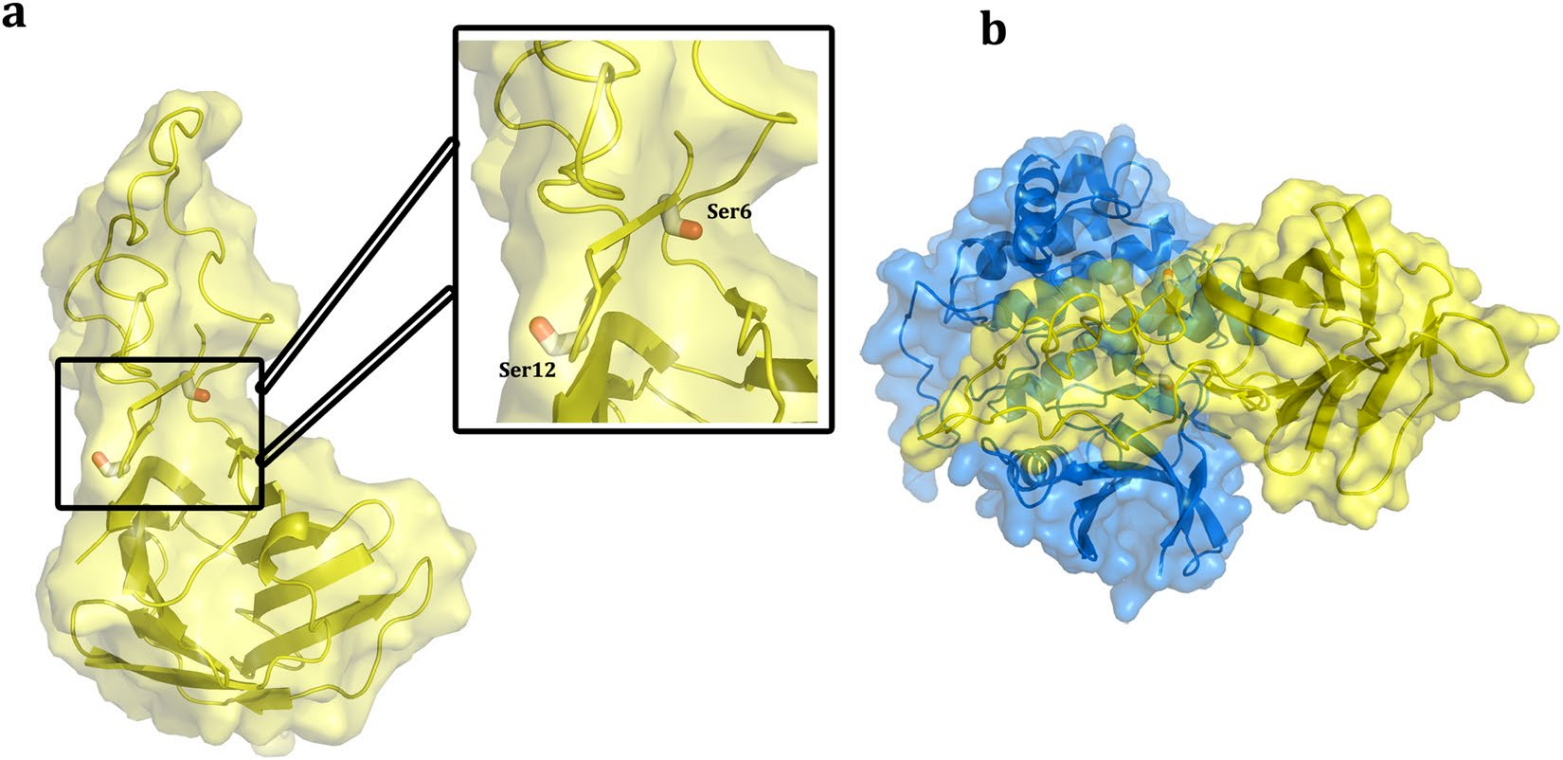
Serine phosphorylation sites. (**a**) Location of the two serine phosphorylation sites. Ser6 and Ser12 in the NTS are easily accessible on the molecular surface of Gal-3 in a pocket between the CRD and the NT. **(b)** Docking of casein kinase I (CK1) onto the structure of Gal-3[NTS/VII-IX] performed with HADDOCK^69^.

This model with its advanced description of the NT structure enables us to examine a mode of presentation of its two sites of phosphorylation. Looking at these target sites for functional post-translational modification, Ser6 and Ser12 are readily accessible in this fixed constellation (Fig. 5a). In more detail, the pocket with Ser6 is a groove that is complementary in shape with the active region of the casein kinase 1 (CK1) (Fig. 5b). The segment comprising repeats VII and VIII not present in the crystallographic structure and modeled by hand in one fixed structure might most likely not represent the entire conformational space, but with its inherent flexibility could adopt the proper conformation to dock to CK1 or undergo a mutual conformational adaptation when the two proteins approach for catalytic phosphorylation. Notably, the same kind of inspection was possible for the sites of tyrosine phosphorylation that reside in the second ordered stretch within the start region of the CRD. Tyr107 and Tyr118 that belong to the CRD are similarly accessible to the solvent and able to be phosphorylated by c-Abl kinase when adopting this crystallographically fixed structure. A further consequence of this model is to give an evidence-based idea of the shape of the full-length Gal-3. To test the validity of the model for actual shape parameters in solution we performed SAXS experiments. In addition to this variant and the full-length Gal-3, we studied an intermediate-length variant with six of the nine collagen-like repeats, i.e. Gal-3[NTS/IV-IX] (for sequence information, please see Supplementary Fig. S1), therefore with a longer NT than Gal-3[NTS/VII-IX].

### Gal-3 Shape: SAXS Analysis vs. Model

**Figure 6:**
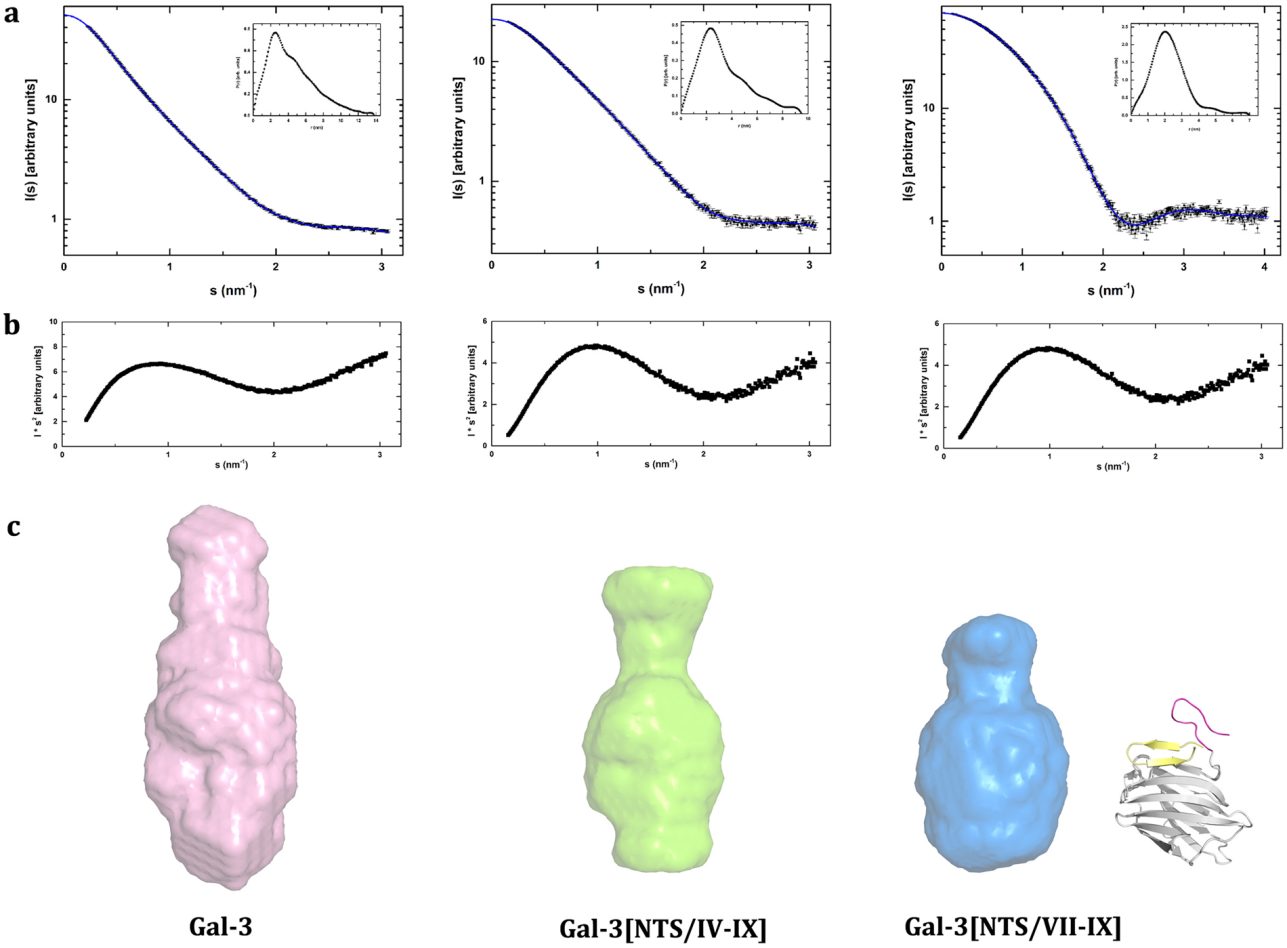
Small-angle X-ray scattering data for Gal-3 and two truncated variants. Scattering curves together with the particle distance distribution P(r) calculated by GNOM (insets) **(a)**, Kratky plots **(b)**, and *ab initio* models **(c)** shown for Gal-3, Gal-3[NTS/IV-IX] and Gal-3[NTS/VII-IX], from left to right. Given on the right side of panel **c**, a comparison between the calculated model and the crystallographic structure of Gal-3[NTS/VII-IX] is presented (see also Fig. 7a).

The scattering curves of the full-length Gal-3 and the two variants with differently shortened NT, i.e. Gal-3[NTS/IV-IX] and Gal-3[NTS/VII-IX], are shown in Fig. 6a,b. Explicitly, the length of the natural NT was thus reduced by deletion of either three or six repeats, whereas the presence of the NTS was maintained. The analysis of the scattering curves gave R_g_ values of 3.54, 2.71 and 1.69 nm as well as maximum dimension (D_max_) values of 13.60, 9.48 and 7.49 nm for the three proteins, respectively. The estimation of the molecular weight is in fair agreement with the respective value of each of the three proteins (Supplementary Table S2). The shape of their distance distribution function (inset of Fig. 6a), together with D_max_ values, indicates that Gal-3 has an elongated shape under these conditions. In comparison, the analysis of the scattering curves (Fig. 6a), together with the Kratky plots (Fig. 6b), came up with Gal-3[NTS/VII-IX] as the most structured protein, followed by a progressive loss of compactness in the Gal-3[NTS/IV-IX] variant and then in full-length Gal-3. *Ab initio* models were generated from the scattering curves (Fig. 6c). They characterize the overall shape of the three proteins (Supplementary Table S2).

**Figure 7:**
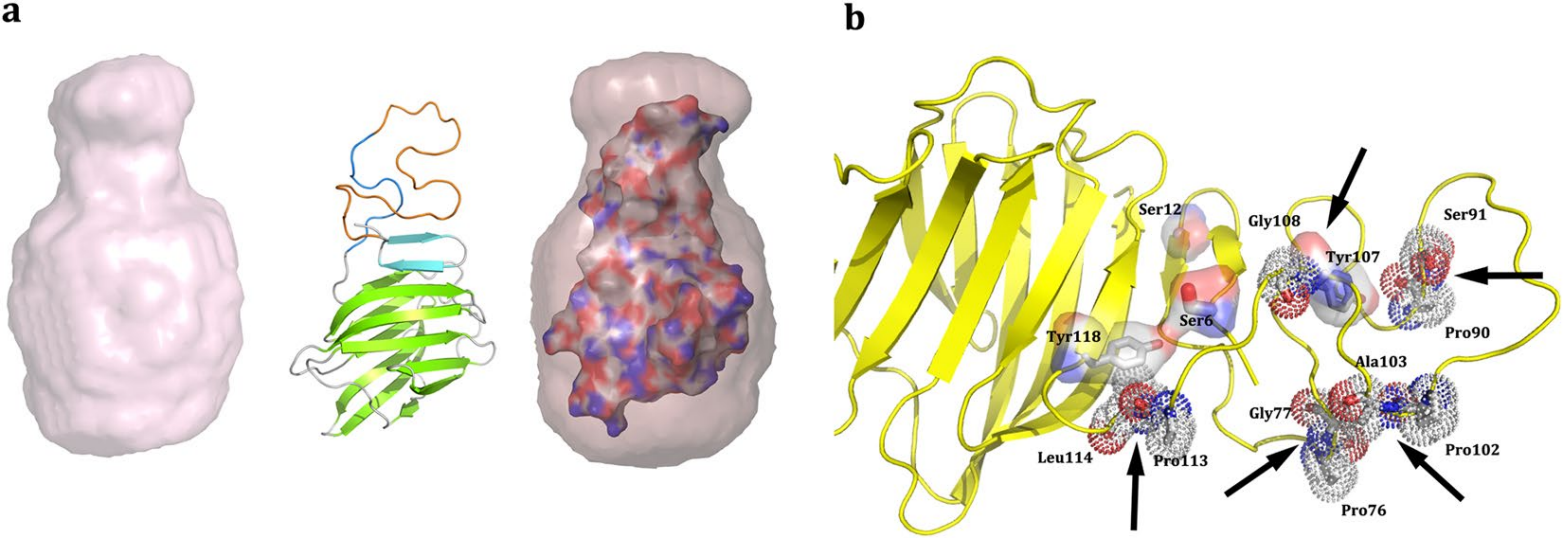
Gal-3[NTS/VII-IX] structure. **(a)** Left, *ab intio* SAXS model. Middle, crystal structure-based model with the missing residues of repeats (orange). Right, superposition of the SAXS and crystallographic models. **(b)** Locations of physiologically relevant proteolytic cleavage sites are indicated by arrows. Also, serines and tyrosines subject to phosphorylation are shown in ball-and-stick mode and in surface representation.

The alignment of the herein reported crystal structure of Gal-3[NTS/VII-IX] with the sphere obtained by these *ab initio* calculations, using the program SUPCOMB^40^ from the ATSAS package^40^, revealed additional information on the SAXS envelope. This allowed us to generate a possible model of the flexible (missing) residues in the crystallographic model. Figure 7a presents a reasonable constellation, where the crystallographic model matches the calculated shape from the SAXS-based data. Considering Gal-3 as substrate for various types of processing, the phosphorylation sites and proteolytic cleavage sites are all accessible to the solvent in this model (Fig. 7b).

## Discussion

Gal-3 has a unique architecture among the members of the galectin family. It underlies this lectin’s capacity to interact with diverse types of counterreceptors (glycans and proteins)^41^. Moreover, this protein harbors sites for post-translational modifications and proteolytic cleavage, the latter acting as a biochemical switch for controlling its capacity for lattice formation^36,38^. In contrast to the 2-fold symmetric dimer organization of most proto-type galectins and the bivalent tandem-repeat-type galectins, the trimodular design also accounts for self-aggregation in the presence of multivalent ligands. Owing to the availability of a growing number of human galectins for testing, functional analysis has moved from work with a single protein to considering their activities as a network. Intriguingly, these efforts are unveiling intense cooperation between galectins. The case of Gal-1 and -3 is a focus of current research, in terms of antagonism^42,43^ and positive cooperation^44^. The competition for the same counterreceptor and the disparity in structural organization of the cross-linked galectin-glycoconjugate complexes, referred to as highly organized (homogeneous) vs heterogeneous aggregates^22^, are assumed to have a context-dependent bearing on cellular responses to galectin binding. These far-reaching physiological implications, with positive or negative consequences on tumor growth regulation or pathogenesis of autoinflammatory disorders^42–44^, explain the enormous interest to clarify the structure of Gal-3 beyond the CRD.

The association of Gal-3 with natural counterreceptors exhibits a different behavior relative to a proto-type galectin. When labeled Gal-3 was probed with surface (microtiter plate well or sensor chip)-immobilized glycoprotein (laminin), binding data revealed positive cooperativity, even for a hamster Gal-3 variant without amino acids 1–93 of the NT^18,45^. Since the collagen-like repeats are endowed with ability for self-aggregation, as shown for example by electron microscopy of rotary shadowed protein preparations^31^ and NMR spectroscopy of ^15^N-labeled protein^33,35^, it is clear that the NT can be a biochemical means toward oligomer formation. The same end was inferred to be reached by mutual recognition between CRDs^28–30,34^, and our crystal structure informs us about a quaternary arrangement via CRD association up to the level of defining the underlying hydrogen-bond pattern. That the nature of the CRD matters to yield aggregation has recently been documented^46^.

In this study, we have been able to obtain crystallized sections of Gal-3 beyond the CRD and to define their structures. The highly dynamic structure, as seen in NMR-based analysis^31,33–35^, can thus adopt conformers that limit the enormous flexibility in solution in distinct sections, allowing crystallization. Evidence for such a restricted conversion between conformers had first been traced in hamster Gal-3 for the region composed of NT and CRD segments^31^. In detail, two parts of the NT could be structurally characterized. This new information enabled us to see the entire CRD, a part of repeat IX and nearly all the NTS, which has so far not been characterized by crystallographic analysis of human Gal-3^37,47,48^. These data provide details on the nature of interactions underlying transient contacts between the CRD and either the NTS or a part of repeat IX. Intramolecular contacts of the collagen-like repeats with the F-face had been inferred to occur previously by NMR spectroscopy of unlabeled and of isotopically labeled full-length and fully truncated Gal-3^31,34–36^.

The information on both regions here identified by crystallography, when implemented into a structural model of Gal-3, offers the opportunity to construct an evidence-based model of full-length Gal-3. The documented possibility for a conformational stabilization of the NTS may be beneficial for its role in cellular compartmentalization^15^ and also for presentation of the two sites for serine phosphorylation. The generated extension of the β-sheet (F–1 and F0 strands) produces a remarkable degree of organization by the interaction of the NTS with the CRD, rationalizing the occurrence of a compact form of Gal-3. Clearly, studies on the other two vertebrate galectins with a N-terminal addition to the CRD, i.e. rat Gal-5 and galectin-related protein^5,49,50^, are now warranted to reveal whether such comparatively short N-terminal extensions will also interact with the CRD.

**Figure 8:**
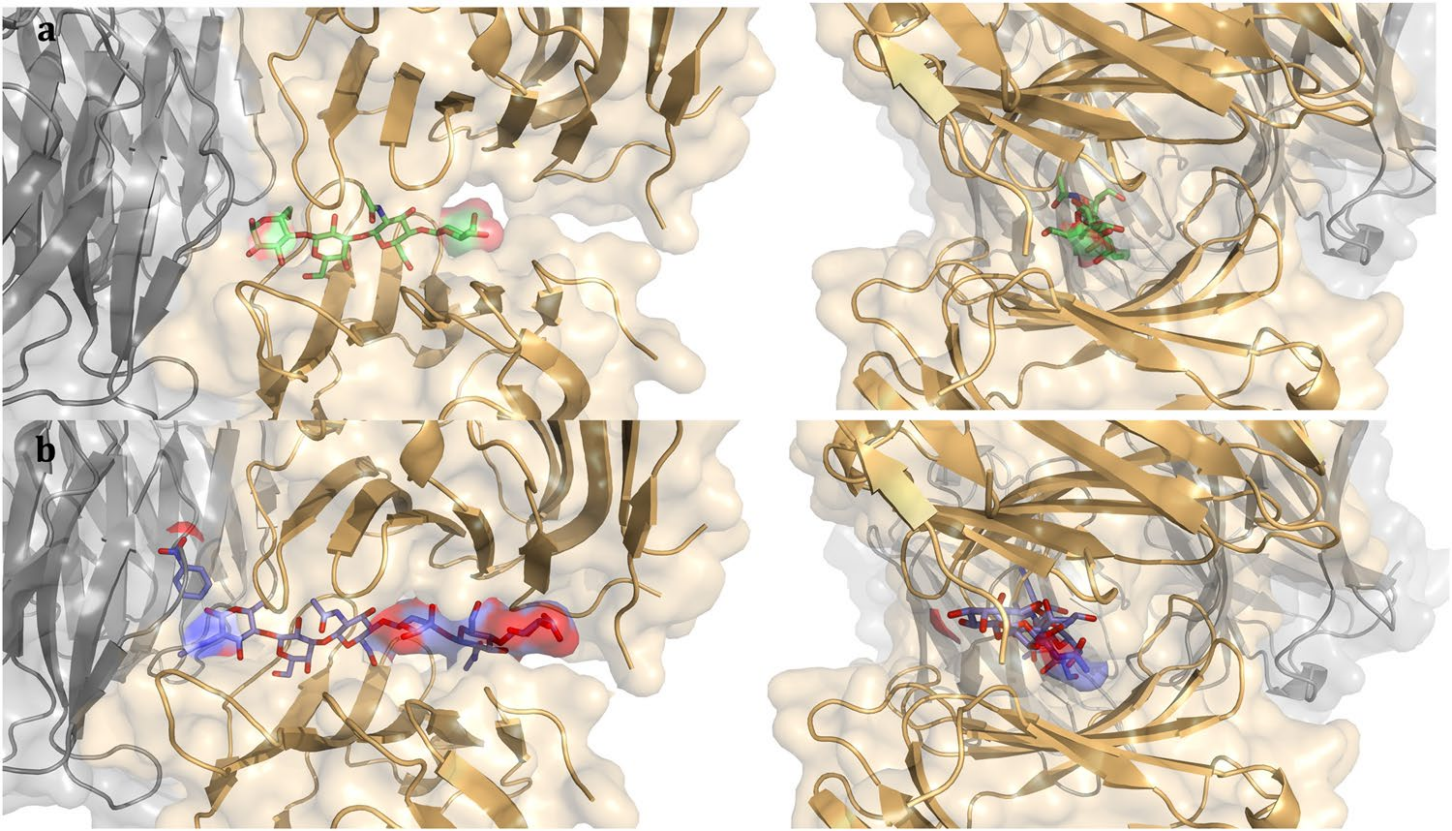
Modelling of positioning of LacNAc-based oligosaccharides in the channel-like tetramer cavity. Docking of the tetrasaccharide of lacto-*N-neo*tetraose (LNnT) **(a)** and of the hexasaccharide of a LacNAc trimer (LN3) **(b)** inside the cavity formed by the protein CRD tetramer using Autodock4^70^. Side (left panels) and front (right panels) views of the models.

When targeting natural glycans, Gal-3 has a high affinity for polyLacNAc chains^51,52^. Crystal structural analysis of Gal-3 in complex with two respective tetrasaccharides (LNT, LNnT) revealed association to the reducing-end galactose unit that explains why Gal-3 can bind to α2,6-sialylated LacNAc oligomers^53,54^. As Fig. 8 illustrates, such a LacNAc-based tetra- or hexasaccharide may act like a string for arraying CRDs. Since contact to the LNnT tetrasaccharide was reported to remove the NT “from the CRD by competition, triggering the release of this N-terminal domain”^33^, LacNAc repeats can favor self-association via the NT and also via the CRD’s F-face^34^, both now fully accessible. A cooperation of these two mechanisms and the oligomer arrangement described herein will likely let Gal-3 acquire the ability to generate more than one topological type of aggregate structure.

In summary, having applied the approach of engineering Gal-3 variants with stepwise-shortened NT^38^, we describe here the first crystallographic evidence of a quaternary structure of a Gal-3 protein stabilized by CRD-CRD contacts. Additionally, a structural representation of two segments of the NT could be defined by crystallographic analysis aided by crystallographic contacts counteracting the high-level flexibility in solution. This feat encourages further work with variant Gal-3 proteins with different NT lengths to relate changes in this parameter to function, in the quest to crack the sugar code^55^. The strategically combined study of hybrids constituted by the NT and a CRD different from that of Gal-3 such as the recently engineered Gal-3NT/8 N protein^56^ and of further glycan ligands such as LacNAc oligomers or 3′-sulfated Lac, which strongly induced glycodendrimersome aggregation by Gal-3^46^, will help to dissect the contributions of the two parts of Gal-3, i.e. NT and CRD, to self-association.

## Methods

### Proteins

Full-length Gal-3 and its two variants with a stepwise truncated NT were obtained by recombinant production in *E. coli* BL21 (DE3)-pLysS cells (Promega, Mannheim, Germany) using pET24a plasmid (Novogen, Darmstadt, Germany), purified by affinity chromatography on lactose-bearing Sepharose 4B obtained by conjugation of ligand to divinyl sulfon-activated resin, then precipitated by addition of ammonium sulfate up to 80% saturation and processed further as given in detail previously^39^. Assessment of molecular integrity and purity by one- and two-dimensional gel electrophoresis, gel filtration and mass spectrometry were done as described^38^.

### Crystallization of Gal-3[NTS/VII-IX]

Crystallization trials were performed at 295 K using the sitting-drop vapor-diffusion method with commercial screening solutions including JBScreen Classic (Jena Bioscience, Jena, Germany), Wizard Classics I–III (Emerald Bio, Bainbridge Island, USA) and Index (Hampton Research, Aliso Viejo, USA) in 96-well sitting-drop plates (Swissci MRC; Molecular Dimensions, Suffolk, England). Drops were set up by mixing equal volumes (0.2 µl) of protein-containing solution at 12 mg/ml and reservoir solution using a Cartesian Honeybee System (Genomic Solutions, Irvine, USA) nano-dispenser robot and equilibrated against 50 µl reservoir solution^39^. However, no crystals were obtained for either full-length Gal-3 or the Gal-3[NTS/IV-IX] variant in any of the conditions tested. Single well-diffracting crystals were obtained in 18% PEG 8 K, 100 mM Tris-HCl (pH 8.5) and 200 mM lithium sulfate. Crystals grew in approximately one month to an average size of 0.15 × 0.15 × 0.10 mm.

### X-ray data collection and structure determination

For data collection, crystals were cryo-protected with a cryo-solution containing the reservoir supplemented with 12.5% (v/v) PEG 400 and flash-frozen in liquid nitrogen. X-Ray data collection experiments were performed at the ALBA Synchrotron (Cerdanyola del Vallès, Spain) BL13 XALOC beamline. The data were indexed and integrated using XDS^57^, scaled and merged using Aimless^58,59^. The structure was solved by molecular replacement using the Gal-3 CRD structure (PDB ID: 1A3K)^37^ with Phaser^60^. The initial model was first refined using Refmac^61^ and alternating manual building with Coot^62^. The final model was obtained by repetitive cycles of refinement; solvent molecules, lactose and sulfate molecules were added automatically and inspected visually for chemically plausible positions. The model was validated and analyzed by MolProbity^63^, figures illustrating protein structure were drawn with PyMOL^64^. Data processing and refinement statistics are listed in Supplementary Table S1. Plot of the average B-factors is shown in Supplementary Fig. S4.

### Small-angle X-ray scattering (SAXS)

SAXS data were collected on BM29 at the European Synchrotron Radiation Facility (ESRF, Grenoble, France) using the BioSAXS robot and a Pilatus 1M detector (Dectris AG, Baden-Daettwil, Switzerland) with synchrotron radiation at a wavelength of λ = 0.1 nm and a sample-detector distance of 2.867 m^65^. Each measurement consisted of 10 frames each of 1 s exposure of a 100 μL sample solution flowing continuously through a 1 mm diameter capillary. Buffer scattering was determined immediately before each measurement of the corresponding protein sample at 269 K. The scattering images obtained were spherically averaged, and the buffer scattering intensities subtracted using in-house software. Protein-containing solutions of Gal-3[NTS/VII-IX], Gal-3[NTS/IV-IX] and full-length Gal-3 were prepared at concentrations of 2, 4, 6, 8 and 10 mg/mL in 20 mM sodium/potassium phosphate buffer at pH 7.0 containing 150 mM NaCl, 4 mM β-mercaptoethanol and 5 mM lactose. Data points affected by aggregation, possibly induced by radiation damage, were excluded. Regularized indirect transforms of the scattering data were performed with the program GNOM^40^ to obtain the radius of gyration (Rg) and P(r) functions of interatomic distances. Three-dimensional bead models that fitted with the scattering data were generated *ab initio* using the program DAMMIF^40^. Multiple runs were performed to generate 20 independent model shapes that were combined and filtered to produce an averaged model using the program DAMAVER^40^.

### Modelling inside the SAXS envelope

The X-ray crystal structure of Gal-3[NTS/VII-IX] was superimposed over the SAXS-defined envelope using SUPCOMB^40^. The connecting segment of about 30 residues between Pro17 and Tyr101, corresponding to the last few NTS residues and repeats VII and VIII of the Gal-3[NTS/VII-IX] variant was analyzed by two secondary-structure prediction servers, i.e. RaptorX^66^ and I-TASSER^67^. The predicted structure was an almost linear and long polypeptide chain. Thus, Coot^62^ was used to build a model interconnecting the visible parts of the NT from the crystal structure. The model was then subjected to different simulated annealing torsion-angle refinement protocols using CNS^68^ with a multi-temperature approach method (3,000 K to 10,000 K) until the model reached convergence at 300 K. The stereochemical quality was then checked with MolProbity^63^ showing reasonable scores, with no bad contacts and 85.8% of the residues in the most favoured regions of the Ramachandran plot. Docking of the structure of the new model for the Gal-3[NTS/VII-IX] variant generated by our data with CK1 kinase was performed using HADDOCK^69^ (HADDOCK score of −95.7 ± 14.0; buried surface area of 3068.6 ± 207.7 Å^2^). Docking of the LacNAc-based saccharides on the CRD tetramer was performed using Autodock4^70^.

## Electronic supplementary material

Supplementary material
