# Supplementary material for: Crystallization of a human galectin-3 variant with two ordered segments in the shortened N-terminal tail
Source: Sci Rep. 2018 Jun 29;8:9835. doi: 10.1038/s41598-018-28235-x (PMC6026190; doi:10.1038/s41598-018-28235-x)
Supplement: Supplementary file 1 — Supplementary material [file 41598_2018_28235_MOESM1_ESM.pdf]

Crystallization of a human galectin-3 variant with two ordered segments in the shortened N-terminal tail

Andrea Flores-Ibarra[1#], Sabine Vértesy[2#], Francisco J. Medrano[1], Hans-Joachim Gabius[2*] and Antonio Romero[1*]

[1]Department of Structural and Chemical Biology, Centro de Investigaciones Biológicas, CSIC, Ramiro de Maeztu 9, 28040 Madrid, Spain

[2]Institute of Physiological Chemistry, Faculty of Veterinary Medicine, Ludwig-Maximilians-University Munich, Veterinärstrabe 13, 80539 Munich, Germany

[#]Contributed equally to this work

[*]Correspondence should be addressed to H.-J.G. (gabius@tiph.vetmed-uni-muenchen.de) or A.R. (romero@cib.csic.es)

Data collection	
Wavelength (Å)	0.9794
Crystal system, Space group	Orthorhombic, $P2_12_12_1$
Unit cell parameters (Å)	a = 93.85, b = 98.19, c = 237.81
Resolution range (Å)*	49.10 – 2.20 (2.32 – 2.20)
Nº of observations	1012467 (147079)
Nº of unique reflections	112248 (16179)
Redundancy	9.0 (9.1)
Completeness (%)	100 (100)
Mean I/σ(I)	11.7 (1.8)
R_{merge}[a]	0.134 (1.233)
R_{meas}[b]	0.142 (1.308)
$CC_{1/2}$[c] (%)	99.7 (55.8)
Wilson B-factor (Å^2)	43.25
Refinement	
Resolution range (Å)	49.50 – 2.20 (2.26 – 2.20)
Nº of working reflections	106537 (7797)
Nº of testing reflections	5607 (413)
Completeness (%)	99.97
R_{work}	0.213 (0.342)
R_{free} #	0.264 (0.350)
Nº of non-H atoms	
Protein	13566
Lactose	276
Sulfate ions	120
Water molecules	128
Average B factors (Å^2)	
Protein	47.15
Lactose	31.14
Sulfate ions	56.26
Water molecules	34.91
RMS deviations	
Bond lengths (Å)	0.014
Bond angles (º)	1.83
Ramachandran plot statistics	
Favoured (%)	96.4
Allowed (%)	3.4
Outliers (%)	0.2
PDB ID code	6FOF

Table S1. X-ray data collection and refinement statistics of Gal-3[NTS/VII-IX]. [a] $R_{merge} = \Sigma_{hkl}\Sigma_i |I_i(hkl) - \langle I(hkl)\rangle| / \Sigma_{hkl}\Sigma_i I_i(hkl)$, [b] $R_{meas} = \Sigma_{hkl} (N-1)^{-1/2} \Sigma_i |I_i(hkl) - \langle I(hkl)\rangle| / \Sigma_{hkl} \Sigma_i I_i(hkl)$, where $I_i(hkl)$ is the intensity measured for the ith reflection and $(I(hkl))$ is the average intensity of all reflections with indices *hkl*. [c] $CC_{1/2}$ is the correlation coefficient between two random half data sets [Karplus, P.A. & Diederichs, K. Linking crystallographic model and data quality. *Science* **336**, 1030-1033 (2012)]. *Values in parentheses are for the highest resolution. # The R_{free} value test set size is 5%.

	P(r) function				*ab initio* modelling
	R_g (nm)	D_{max} (nm)	Est. Mw (kDa)	Mw (kDa)	Discrepancy (χ^2)
Gal-3	3.54 ± 0.31	13.60 ± 1.58	26.21 ± 1.43	26.15	5.05 ± 1.37
Gal-3[NTS/IV-IX]	2.71 ± 0.14	9.48 ± 0.64	22.95 ± 2.22	23.55	1.13 ± 0.01
Gal-3[NTS/VII-IX]	1.69 ± 0.06	7.49 ± 0.51	21.30 ± 3.14	21.32	1.26 ± 0.09

Table S2. Small angle X-ray scattering data statistics

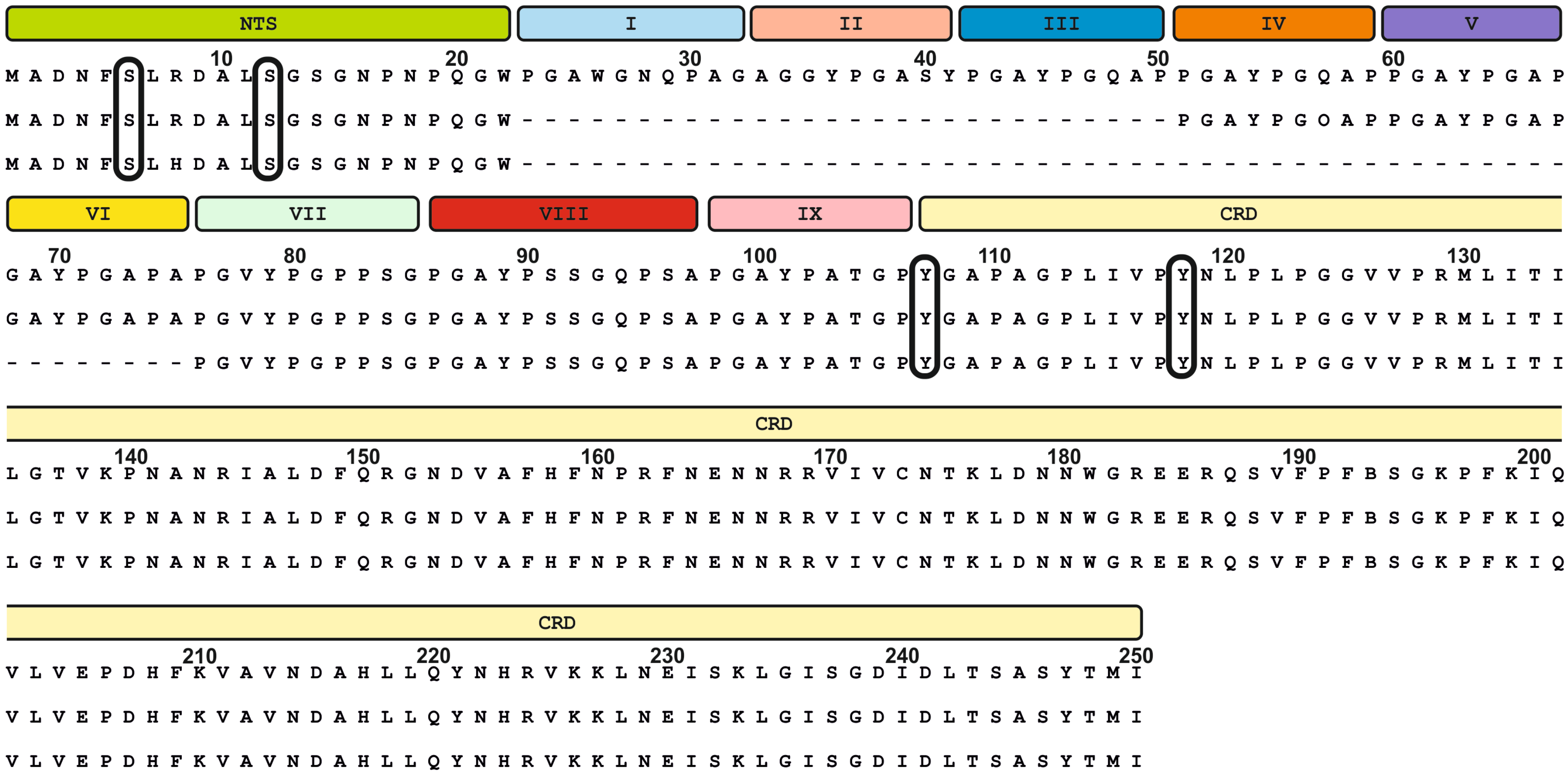

Figure S1. Sequences of full-length Gal-3 (FL Gal-3) and its two variants based on deletion of either three (Gal-3[NTS/IV-IX]) or six (Gal-3[NTS/VII-IX]) collagen-like repeats.

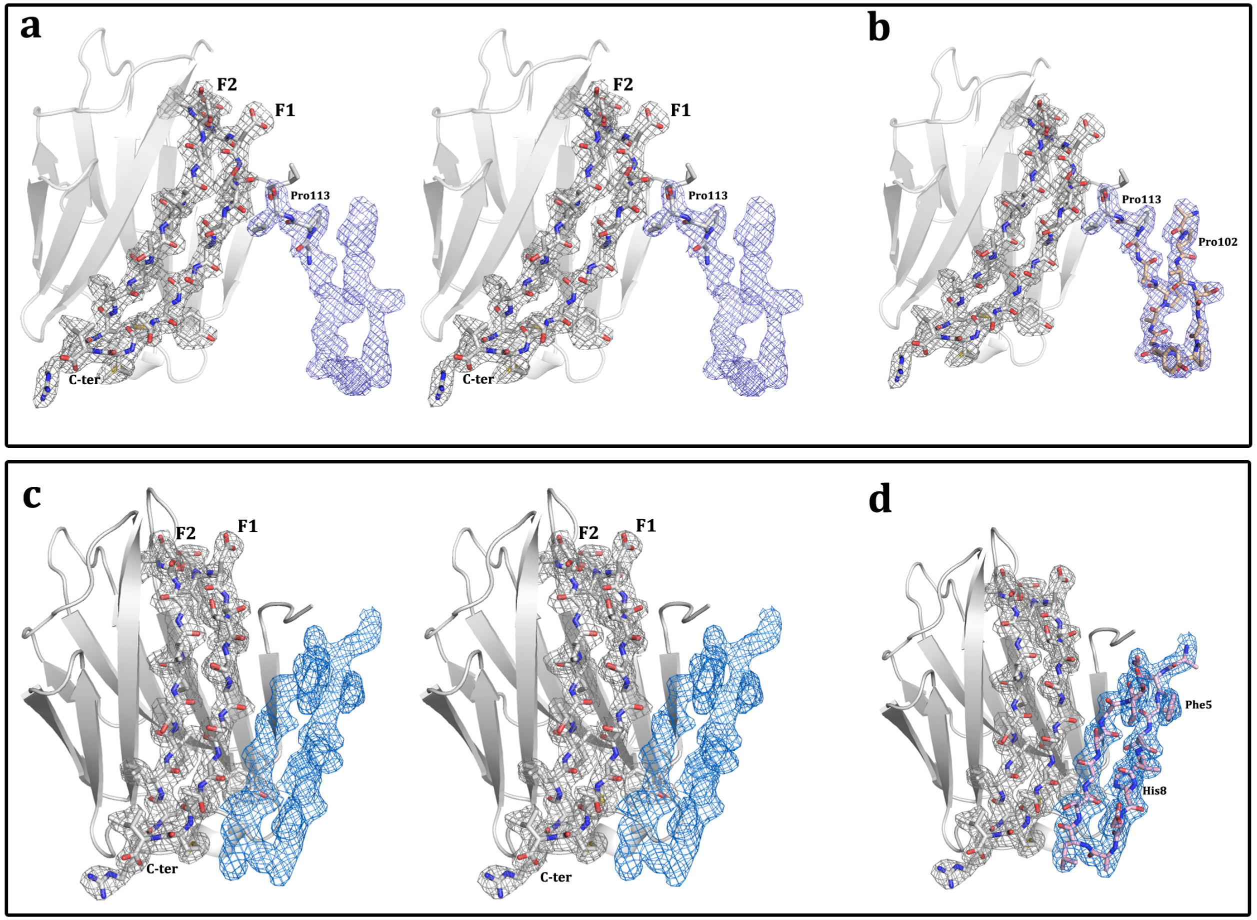

Figure S2. **Crystallographic structure of two regions of the NT. (a)** Stereo view of the *2mFo-DFc* electron density maps contoured at 1σ of the repeat IX linked to the CRD. **(b)** Building of residue positioning inside the electron density of the repeat IX linked to the CRD. **(c)** Stereo view of the *2mFo-DFc* electron density maps contoured at 1σ of the NTS region. **(d)** Positioning of the residues inside the electron density with the two fingerprint-like rings of His8 and Phe5.

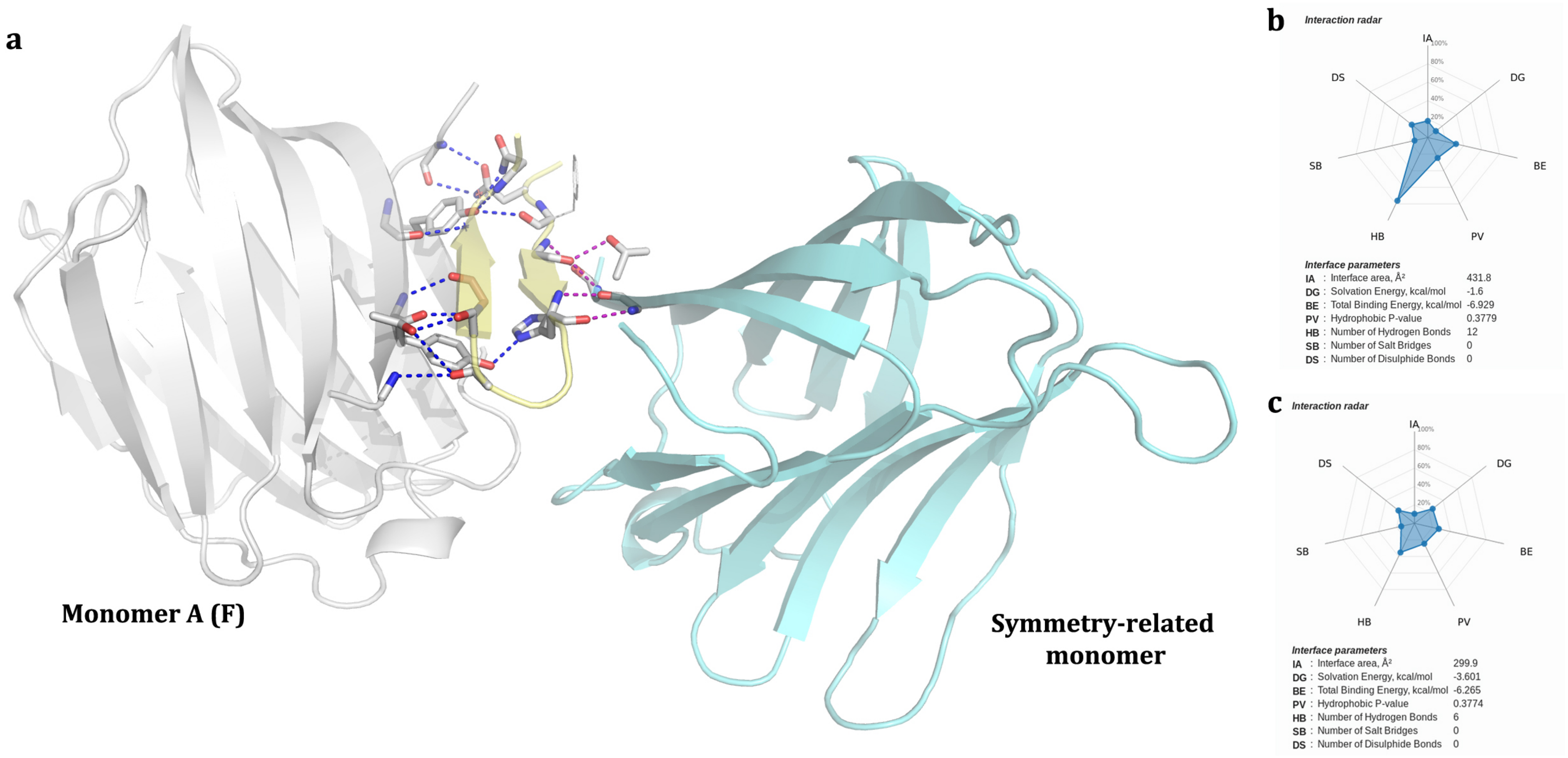

Figure S3. Snapshot of the N-terminal region. **(a)** The NTS appears in the crystal structure wedge between the F-faces of two separate molecules, one of them a symmetry-related molecule. Hydrogen bonds are depicted in blue (monomer A or F) or in purple (with a symmetry-related monomer). PISA interaction radar scoring the macromolecular interfaces of the NTS with monomer A (F) **(b)** and with a symmetry-related monomer **(c)**.

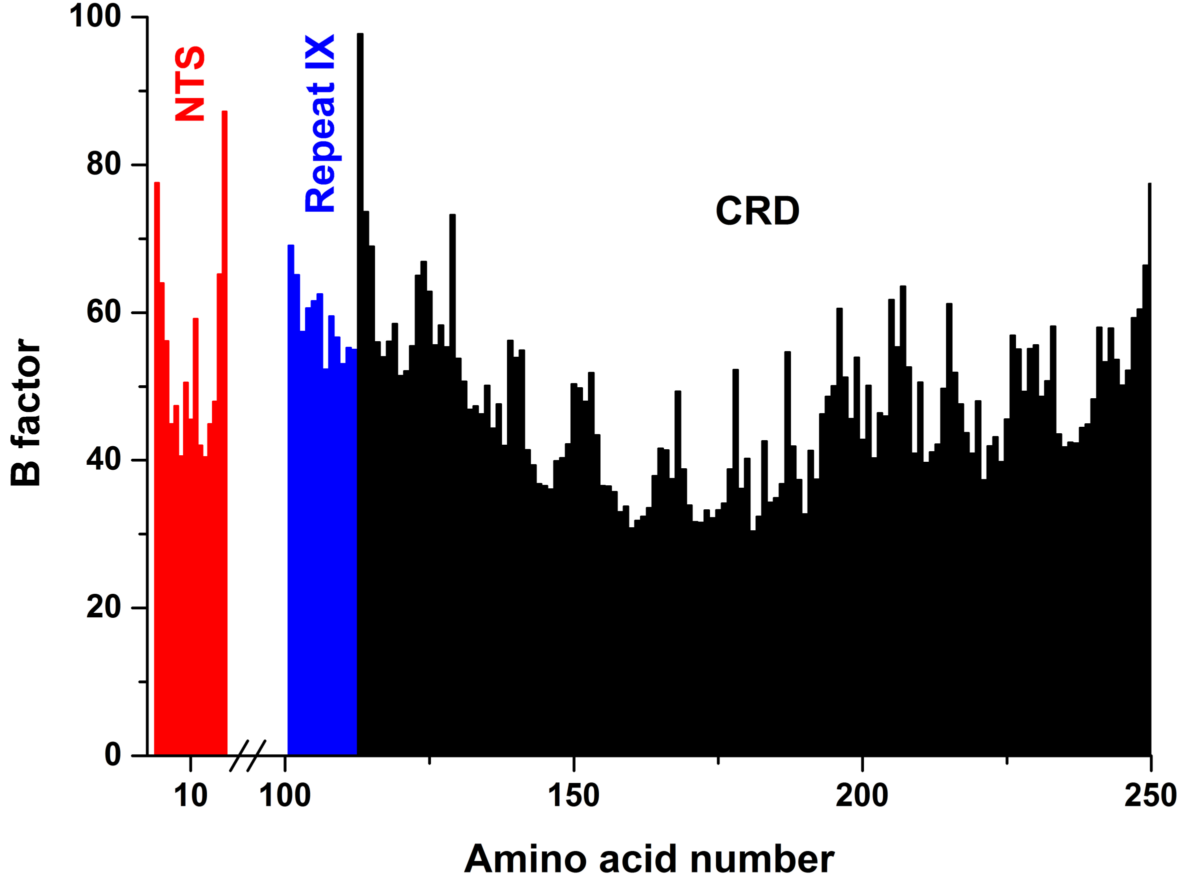

Figure S4. Average B-factor plot by amino acids of the Gal-3[NTS/VII-IX] structure.
